# Hepatic Expression of Detoxification Enzymes Is Decreased in Human Obstructive Cholestasis Due to Gallstone Biliary Obstruction

**DOI:** 10.1371/journal.pone.0120055

**Published:** 2015-03-23

**Authors:** Jin Chai, Xinchan Feng, Liangjun Zhang, Sheng Chen, Ying Cheng, Xiaochong He, Yingxue Yang, Yu He, Huaizhi Wang, Rongquan Wang, Wensheng Chen

**Affiliations:** 1 Department of Gastroenterology, Southwest Hospital, Third Military Medical University, Chongqing, P.R. China; 2 Department of Pediatrics, Southwest Hospital, Third Military Medical University, Chongqing, P.R. China; 3 School of Nursing, Third Military Medical University, Chongqing, P.R. China; 4 Department of Gastroenterology, West China Hospital, Sichuan University, Chengdu, Sichuan, P.R. China; 5 Institute of Hepatobiliary Surgery, Southwest Hospital, Third Military Medical University, Chongqing, P.R. China; University of Texas Health Science Center, UNITED STATES

## Abstract

**Background & Aims:**

Levels of bile acid metabolic enzymes and membrane transporters have been reported to change in cholestasis. These alterations (e.g. CYP7A1 repression and MRP4 induction) are thought to be adaptive responses that attenuate cholestatic liver injury. However, the molecular mechanisms of these adaptive responses in human obstructive cholestasis due to gallstone biliary obstruction remain unclear.

**Methods:**

We collected liver samples from cholestatic patients with biliary obstruction due to gallstones and from control patients without liver disease (n = 22 per group). The expression levels of bile acid synthetic and detoxification enzymes, membrane transporters, and the related nuclear receptors and transcriptional factors were measured.

**Results:**

The levels of bile acid synthetic enzymes, CYP7B1 and CYP8B1, and the detoxification enzyme CYP2B6 were increased in cholestatic livers by 2.4-fold, 2.8-fold, and 1.9-fold, respectively (*p*<0.05). Conversely, the expression levels of liver detoxification enzymes, UGT2B4/7, SULT2A1, GSTA1-4, and GSTM1-4, were reduced by approximately 50% (p<0.05) in human obstructive cholestasis. The levels of membrane transporters, OSTβ and OCT1, were increased 10.4-fold and 1.8-fold, respectively, (*p*<0.05), whereas those of OSTα, ABCG2 and ABCG8 were all decreased by approximately 40%, (*p*<0.05) in human cholestatic livers. Hepatic nuclear receptors, VDR, HNF4α, RXRα and RARα, were induced (approximately 2.0-fold, (*p*<0.05) whereas FXR levels were markedly reduced to 44% of control, (*p*<0.05) in human obstructive cholestasis. There was a significantly positive correlation between the reduction in FXR mRNA and UGT2B4/7, SULT2A1, GSTA1, ABCG2/8 mRNA levels in livers of obstructive cholestatic patients (*p*<0.05).

**Conclusions:**

The levels of hepatic detoxification enzymes were significantly decreased in human obstructive cholestasis, and these decreases were positively associated with a marked reduction of FXR levels. These findings are consistent with impaired detoxification ability in human obstructive cholestasis.

## Introduction

The expression levels of hepatic membrane transporters and bile acid metabolic enzymes have been shown to be significantly altered in cholestasis [1–5]. Most of these changes are thought to be an “anti-cholestasis” adaptive response in rodents [5–10]. For example, bile acid efflux transporter multidrug resistance protein 4 (Mrp4) deficiency worsened liver injury and decreased plasma bile acid levels in bile duct-ligated mice. However, the expression of MRP4/Mrp4 at the basolateral membrane of cholestatic hepatocytes was markedly induced in human and rodent cholestasis [2–4, 11–13]. Inhibition of bile acid synthetic enzymes, cytochrome P450 (Cyp)7a1 and Cyp7b1, and induction of detoxification enzymes, Cyp3a11, UDP-glucuronosyltransferase(Ugt)2b and soluble sulfotransferases(Sult)2a1, that contribute to decrease bile acid synthesis and toxicity have been observed in cholestatic rodents [1–5, 14]. However, only CYP7A1 repression was detected in human obstructive cholestasis and primary biliary cirrhosis (PBC) [6, 10, 12]. In contrast, the induced expression of CYP8B1 was observed in extrahepatic cholestasis caused by pancreatic tumors [12]. These data suggested that the molecular mechanism of adaptive response may vary depending on the organism and type of cholestasis. Whether a similar adaptive response is coordinately regulated in human obstructive cholestasis originating from gallstone blockage of bile ducts remains unclear.

Hepatic nuclear receptors (NRs) and hepatocyte-enriched transcription factors (TFs) are modulators of the expression levels of bile acid synthetic and detoxification enzymes and membrane transporters [2, 3, 7, 13–15]. For example, the bile salt receptor Fxr (farnesoid-X receptor) represses the expression of the bile acid synthetic enzyme, Cyp7a1, and the influx transporter, Na^+^/taurocholate cotransporter (Ntcp), in rodent cholestasis [2, 3, 14]. This occurs by activation of a short heterodimer partner (SHP), and induction of the expression of bile acid efflux transporter, organic solute transporter alpha and beta (Ostα/β), and detoxification enzymes, Cyp3a11 and Ugt2b, in cholestatic rodent liver [2–6, 9, 14–16]. In addition, other nuclear receptors, e.g. retinoid X receptor (RXRα/Rxα) and retinoic acid receptor (Rarα), and transcriptional factors (e.g NRF2, and AHR) are also involved in the adaptive response in cholestatic rodents and hepatocytes [3, 6, 14, 16–22]. However, the exact nature of these changes and the functional roles of these modulators in human obstructive cholestasis due to gallstone biliary obstruction have not yet been fully elucidated.

The aim of this study was to compare control patients and obstructive cholestatic patients with gallstone biliary obstruction, by measuring the expression levels of adaptive response genes and the relevant nuclear receptors and transcriptional factors.

## Materials and Methods

### Patients and liver sample collection

All human liver samples were collected from Southwest Hospital (Chongqing, China). The research was carried out in accordance with the Declaration of Helsinki (2008) of the World Medical Association, and approved by the Southwest Hospital Institutional Ethics Review Board. Written informed consent was obtained from all participants. Liver samples were acquired from control patients by liver biopsy for exclusion of liver diseases or staging of hematologic disorders (n = 7). Samples were also obtained from patients undergoing resection of liver metastases (n = 15; 7 hematologic malignancy, 6 colorectal, 7 colonic, and 2 rectal metastases). Cholestatic liver samples (n = 22) were surgically resected from patients with obstructive cholestasis caused by biliary stones originating from the intrahepatic bile duct and common bile duct. Neither ursodeoxycholic acid nor other preoperative therapies were administered. Most of the liver specimens from control patients (n = 17) and cholestatic patients (n = 22) were analyzed previously [6]. Five control liver specimens were new samples from liver biopsies performed for exclusion of liver disease in this study. The isolated liver samples were immediately cut into small pieces and stored in liquid nitrogen. Gender was not a factor involved in altering adaptive response gene expression in cholestatic patients. Biochemical characteristics of patients are described as previously [6].

### RNA extraction and real-time reverse transcription polymerase chain reaction (qPCR)

Samples of liver tissue (100 mg) frozen in liquid nitrogen were ground and subsequently used for extraction of total RNA with Trizol according to instructions of the vendor (Invitrogen; San Diego, CA, USA). The cDNA of patient liver samples was prepared and real-time qPCR was performed as described previously [6]. The primers used in this study are given in S1 Table. OSTα, OSTβ, GAPDH and β-actin primers were used as described [6, 23]. GAPDH and β-actin were used as references for normalizing data, and real-time PCR amplification efficiency of target genes was considered, when using CFX manager 2.0 for data analysis (Bio-Rad).

### Western blot analysis

Total membranes, protein and nuclear extracts were prepared and protein concentrations were determined as described previously [6, 7]. The dilution of primary antibodies were as follows: CYP7B1 (1:2000), CYP8B1 (1:2000), CYP27A1 (1:2000), CYP3A4 (1:4000), UGT2B (1:4000), SULT2A1 (1:2000), GSTA1 (1:1000), GSTM2 (1:1000), OSTα (1:4000), OCT1 (1:2000), ABCG5 (1:3000), ABCG8 (1:2000), VDR (1:1000), RARα (1:1600), HNF1α (1:2000), HNF4α (1:2000), and LXR (1:1000) (Santa Cruz Biotechnology, Santa Cruz, CA), CYP2B6 (1:2000) (OriGene, Rockville, MD), GSTA2 (1:4000) (GeneTex, Irvine, CA), GSTA3 (1:1000), GSTA4 (1:1000), GSTM1 (1:1000), GSTM3 (1:1000), GSM4 (1:1000), PPARα (1:1000), and AHR (1:1000) (Proteintech Group, Chicago, IL, USA), OSTβ (1:500) (Sigma-Aldrich Chemical Co., St Louis, MO), ABCG2 (1:2000), FXR (1:10,000), SHP (1:1000), HNF3β (1:10,000), and NRF2 (1:10,000) (Abcam, Cambridge, MA). GAPDH (1:40,000) (Abcam) and SH-PTP1 (1:1600) (Santa Cruz) were used for normalizing data.

### Immunofluorescence analysis

Immunofluorescence (IF) microscopy, combining the antibodies with OCT1 (1:50), OSTα (1:100), OSTβ (1:20), ABCG2 (1:50), ABCG8 (1:50), VDR (1:50), HNF4α (1:50), RARα (1:50) and FXR (1:100), was performed as previously described [6, 7].

### Statistical analysis

All data were analyzed by GraphPad Prism 6.01 (GraphPad Software Inc., San Diego, CA) using the independent-samples Student’s *t*-test (two-tailed) and results were expressed as the means ± standard deviations (SDs). The correlation among FXR, UGT2B4/7, SULT2A1, GSTA1, and ABCG2/8 mRNA was assessed by the Pearson’s correlation test. A value of *p* < 0.05 was considered significant.

## Results

### Expression levels of bile acid synthetic enzymes CYP7B1 and CYP8B1 in obstructive cholestatic patients

**Fig 1:**
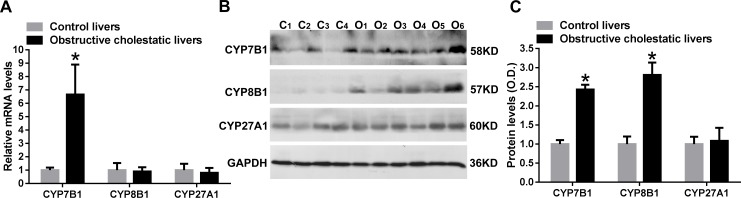
Levels of bile acid synthetic enzymes, CYP7B1 and CYP8B1, in liver samples of patients with obstructive cholestasis. A) Real-time qPCR analysis of CYP7B1, CYP8B1 and CYP27A1 mRNA levels in liver tissues of patients with obstructive cholestasis and controls. B) and C) Western blot analysis of CYP7B1, CYP8B1 and CYP27A1 protein levels in cholestatic livers and controls. Data were analyzed using independent samples *t* test. *: *p* < 0.01 vs. controls. *C*, controls, *n = 22*; *O*, obstructive cholestasis liver samples, *n = 22*.

We have previously reported that a marked reduction in expression of the classical pathway enzyme, CYP7A1, was observed in obstructive cholestatic patients [6]. In contrast, the mRNA and protein levels of the alternative pathway enzyme, CYP7B1, were found to be significantly increased by 6.7-fold and 2.4-fold, respectively, (*p*<0.01) in liver samples of patients with obstructive cholestasis compared to controls, Fig. 1A, B and C). Furthermore, the protein levels of CYP8B1 were increased by 2.8-fold (*p*<0.01), but the mRNA levels were not changed in the liver of obstructive cholestatic patients (Fig. 1A, B and C). However, we observed that the mRNA and protein levels of CYP27A1 were not significantly altered in the livers of patients with obstructive cholestasis, when compared to controls (Fig. 1A, B and C).

### Reduction of levels of hepatic detoxification enzymes in patients with obstructive cholestasis

**Fig 2:**
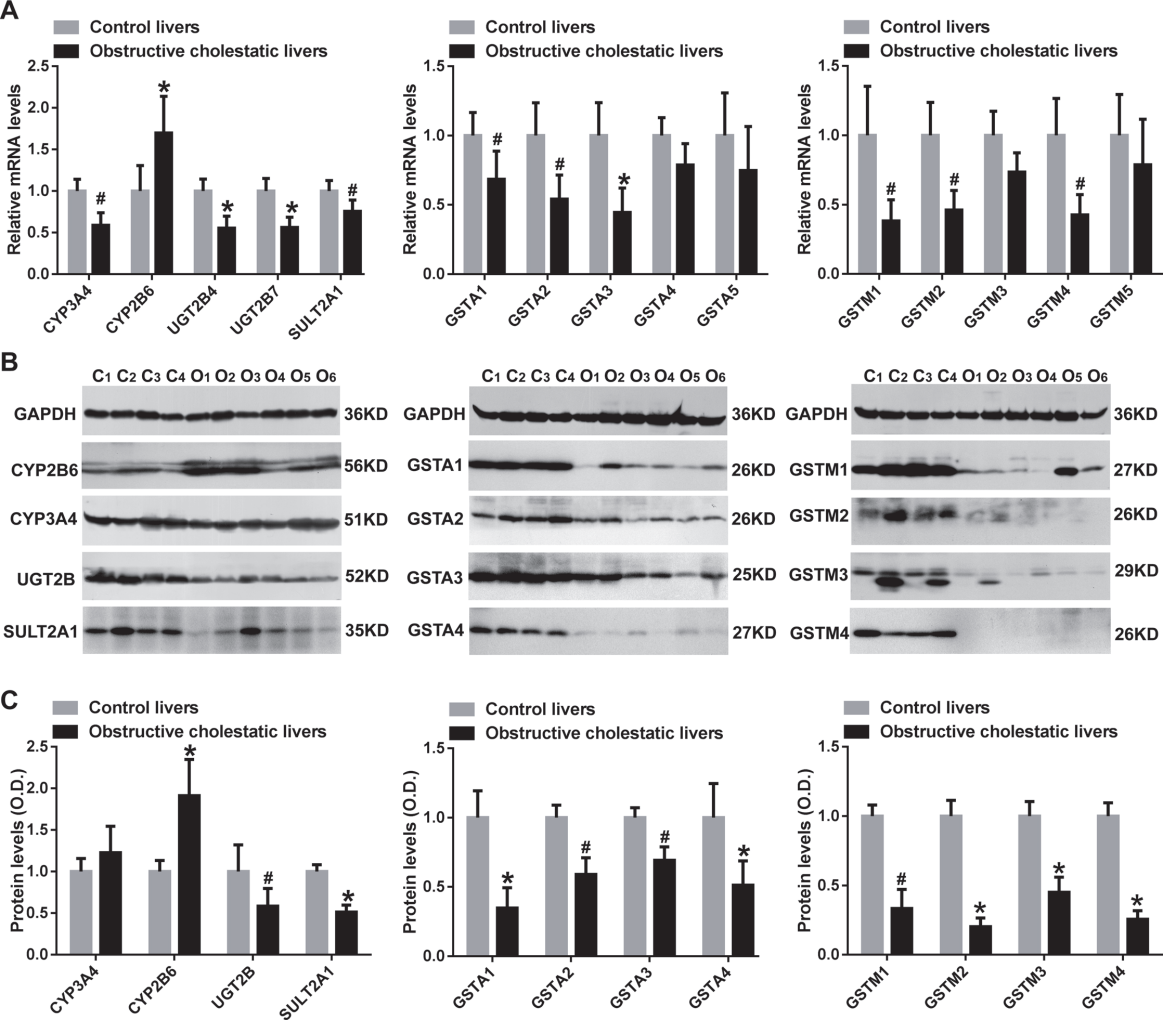
The levels of detoxification enzymes in liver samples of patients with obstructive cholestasis. A) Real-time qPCR analysis of detoxification enzymes CYP2B6, CYP3A4, UGT2B4, UGT2B7, SULT2A1, GSTA1-A5 and GSTM1-M5 mRNA levels in cholestatic livers and controls of patients. B) Representative western blots for CYP2B6, CYP3A4, UGT2B, SULT2A1, GSTA1-GSTA4 and GSTM1-GSTM4 protein levels in liver samples of patients with obstructive cholestasis and (C) corresponding densitometry (% of control group, n = 22 for each group). *: *p* < 0.01 and ^#^: *p* < 0.05 versus controls. *C*, controls, *O*, obstructive cholestasis liver samples.

Our results demonstrated that CYP3A4, UGT2B4, UGT2B7, and SULT2A1 mRNA levels in liver samples of patients with obstructive cholestasis were decreased to 59%, 55%, 56% and 75% of control liver samples, respectively (*p*<0.05 for all, Fig. 2A). Western blot data also indicated that both UGT2B and SULT2A1 protein levels were significantly reduced to 58% and 51% of the control group (*p*<0.01 and *p*<0.05, respectively). In contrast, CYP2B6 mRNA and protein levels were significantly increased in the livers of patients with obstructive cholestasis (1.7-fold and 1.9-fold, respectively, *p<*0.05), compared to the controls (Fig. 2A-C). However, CYP3A4 protein levels were not changed in human cholestatic livers (Fig. 2B and 2C). Moreover, we also determined the expression of GSTs in human obstructive cholestasis. Real-time qPCR revealed that liver GSTA1, GSTA2, and GSTA3 mRNA levels decreased to 69%, 54% and 45% of the control group, respectively (*p*<0.05 for all), while the levels of GSTA4 and GSTA5 mRNA were unaltered (Fig. 2A). As shown in Fig. 2B, Western blot analysis demonstrated that hepatic GSTA1, GSTA2, GSTA3, and GSTA4 protein levels in patients with obstructive cholestasis were reduced to 35%, 59%, 69% and 51% of control, respectively (*p*<0.05 for all). Hepatic mRNA levels of GSTM1, GSTM2, and GSTM4 in patients with cholestasis were decreased to 38%, 46%, and 43% of control, respectively (*p*<0.05 for all), while GSTM3 and GSTM4 mRNA levels were not significantly changed (Fig. 2A). Reduced GSTM1, GSTM2, GSTM3 and GSTM4 protein levels measured by Western blot analysis in human cholestatic livers were 33%, 20%, 45% and 26% of controls, respectively (*p*<0.05 for all) (Fig. 2B and 2C).

### Up-regulation of basolateral membrane transporters, OSTβ and OCT1, and down-regulation of OSTα in human cholestatic liver samples

**Fig 3:**
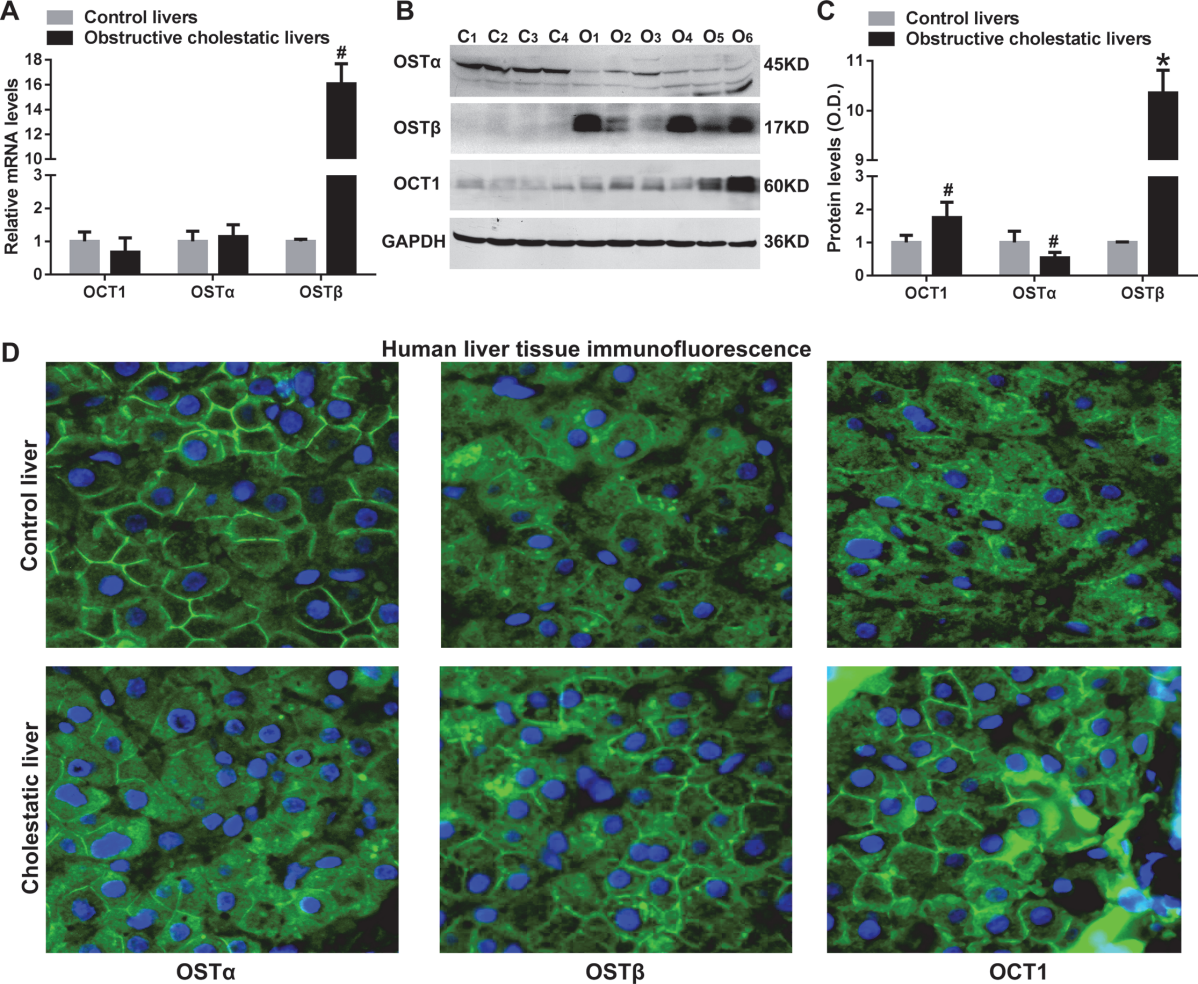
OSTα/β and OCT1 levels at the basolateral membrane of cholestatic hepatocytes of patients. (A) Real-time qPCR analysis of mRNA levels of efflux transporters OSTα, OSTβ and OCT1 in liver samples of patients with obstructive cholestasis. (B) Representative western blots for OSTα, OSTβ and OCT1 and (C) corresponding densitometry (n = 22 for each group). (D) IF labeling of OSTα, OSTβ and OCT1 protein (green) in the liver of patients with obstructive cholestasis and controls. Nuclei of hepatocytes from liver samples were stained with DAPI (blue). *C*, controls; *O*, obstructive cholestasis liver samples. *p < 0.*01*; ^#^ *p <0*.*05* versus controls. DAPI, 4′, 6-diamidino-2-phenylindole.

The OSTβ mRNA levels were increased 16-fold when compared to controls (p<0.05), whereas the mRNA levels of OSTα and OCT1 were not significantly changed (Fig. 3A). Western blots showed that hepatic OSTβ and OCT1 protein levels were significantly increased by 10.4-fold, *p*<0.01 and 1.8-fold, (*p*<0.05, respectively, Fig. 3B and 3C). In contrast, the OSTα protein levels were decreased to 54% of the control group in obstructive cholestatic patients (*p*<0.05, Fig. 3B and 3C). Moreover, up-regulation of OSTβ and OCT1 proteins and down-regulation of OSTα protein levels at the basolateral membrane of hepatocytes in cholestatic patients were further confirmed by immunofluorescence staining and microscopy (Fig. 3D).

### Decreased expression of canalicular membrane transporters ABCG2 and ABCG8 in liver samples of obstructive cholestatic patients

**Fig 4:**
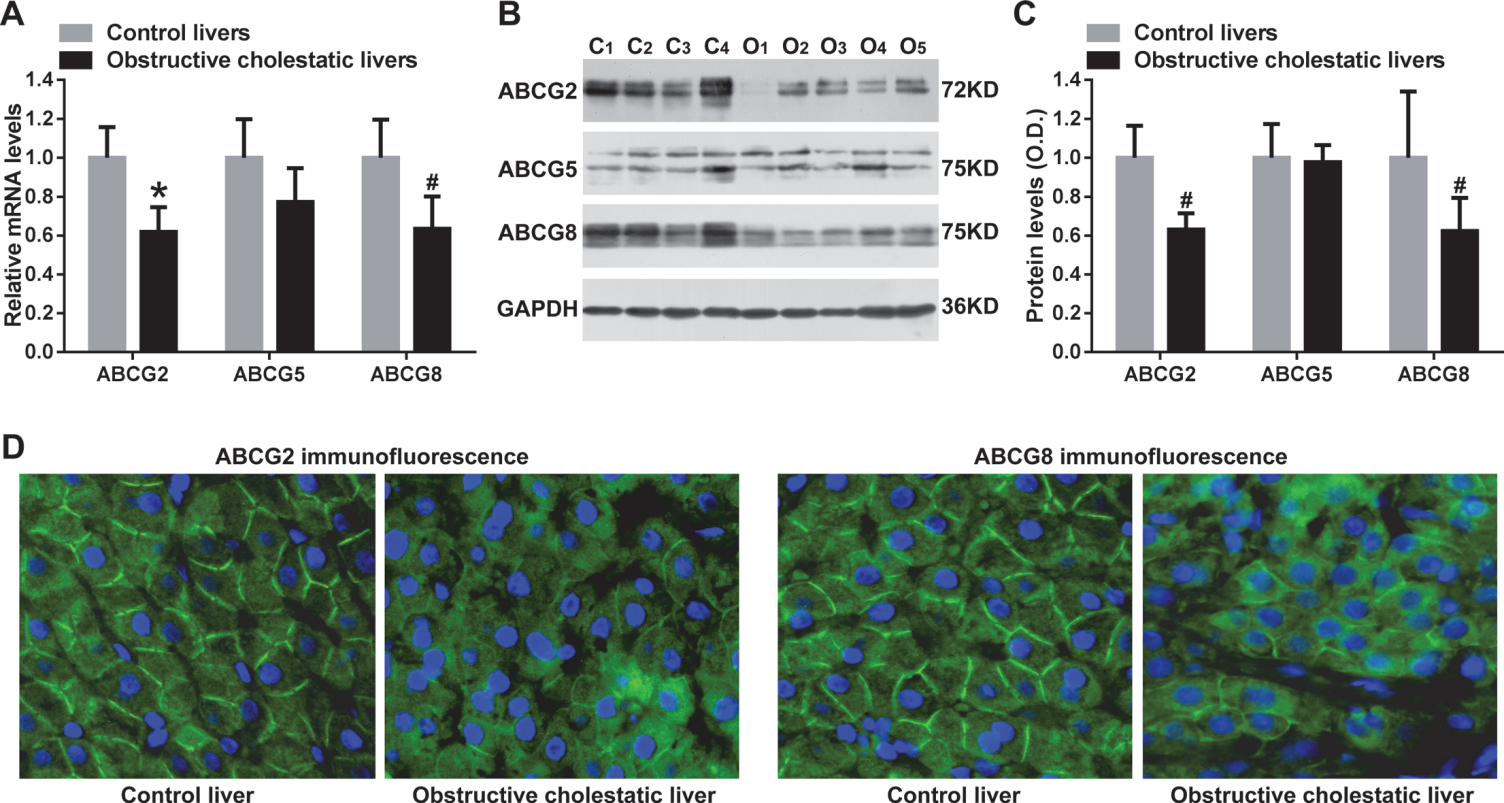
Hepatic canalicular membrane transporters ABCG2 and ABCG8 expression in human obstructive cholestasis. (A) Real-time qPCR analysis of mRNA levels of canalicular membrane transporters ABCG2, ABCG5 and ABCG8 (% of control group, n = 22 for each group). (B) Representative Western Blotting for ABCG2, ABCG5 and ABCG8 and (C) their corresponding densitometry (% of control group, n = 22 for each group). (D) IF labeling of ABCG2 and ABCG8 protein (green) in the livers from patients with obstructive cholestasis and controls. Nuclei of hepatocytes from liver samples were stained with DAPI (blue). *C*, controls; *O*, obstructive cholestasis liver samples. *p < 0.*01*; ^#^ *p <0*.*05* versus controls. DAPI, 4′, 6-diamidino-2-phenylindole.

Real time qPCR analysis showed that canalicular membrane transporters ABCG2 and ABCG8 mRNA levels were significantly reduced in the livers of cholestatic patients (38%, *p*<0.01, and 36% *p*<0.05, of controls, respectively, Fig. 4A). Western blot analysis confirmed that the protein levels of ABCG2 and ABCG8 were decreased to 63% and 62% those of control human cholestatic liver samples, respectively (*p*<0.05 for all, Fig. 4B). Reduced protein levels of canalicular membrane ABCG2 and ABCG8 in obstructive cholestatic hepatocytes of patients were further confirmed by immunofluorescent labeling with anti-ABCG2 and anti-ABCG8 antibodies (Fig. 4C). However, the levels of ABCG5 mRNA and protein were not altered in liver samples of patients with obstructive cholestasis compared to control (Fig. 4A, B and C).

### Significant reduction in levels of the bile acid receptor FXR in livers of obstructive cholestatic patients

**Fig 5:**
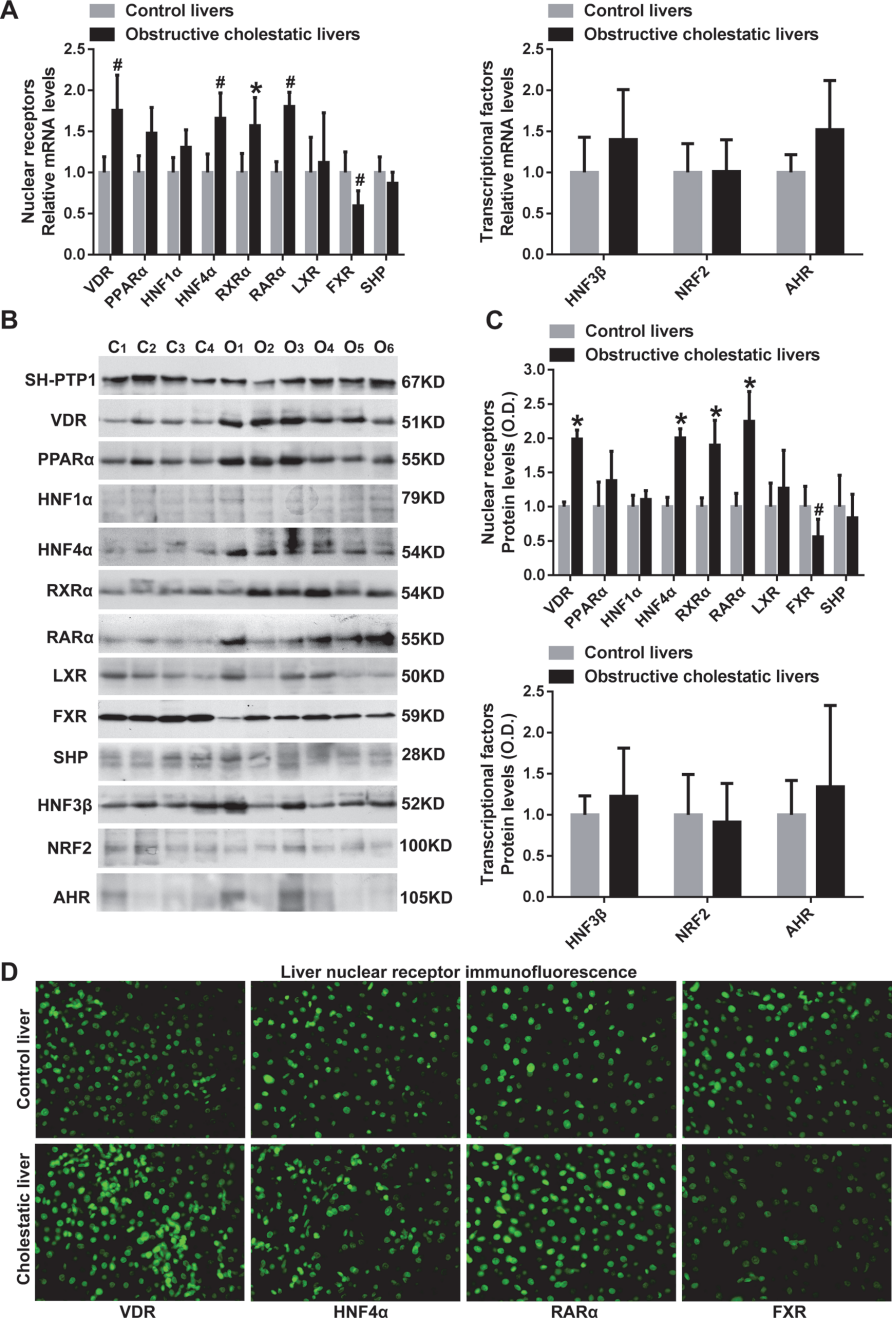
Nuclear receptors, VDR, HNF4α, RARα, and FXR levels in liver samples from patients with obstructive cholestasis. Real-time qPCR analysis of nuclear receptors mRNA levels (A) VDR, PPARα, HNF1α, HNF4α, RXRα, RARα, LXR, FXR, and SHP, and transcriptional factors HNF3β, NRF2 and AHR (n = 22 for each group). (B) Representative western blot for VDR, PPARα, HNF1α, HNF4α, RXRα, RARα, LXR, FXR, SHP, and HNF3β, NRF2 and AHR and (C) their corresponding densitometry (% of control group, n = 22 for each group). (D) IF labeling of VDR, HNF4α, RARα, and FXR protein (green) in the liver of patients with obstructive cholestasis and controls. *C*, controls; *O*, obstructive cholestasis liver samples. *p < 0.*01*; ^#^ *p <0*.*05* versus controls.

We demonstrated that hepatic mRNA levels of nuclear receptors VDR, HNF4α, RXRα and RARα were significantly increased in cholestatic patients 1.8-fold, 1.7-fold, 1.6-fold and 1.8-fold, respectively, (*p*<0.05 for all), when compared to controls (Fig. 5A). PPARα mRNA levels expression also tended to be increased by 1.5-fold (*p* = 0.05, Fig. 5A). In contrast, FXR mRNA levels in cholestatic hepatocytes were decreased to 59% of those of control samples (*p*<0.05, Fig. 5A). In contrast, the mRNA levels of nuclear receptors HNF1α, LXR and SHP and transcription factors HNF3β, NRF2 and AHR were not changed (Fig. 5A). Western blot analysis showed that the levels of VDR, HNF4α, RXRα and RARα proteins were increased in liver samples of patients with obstructive cholestasis 2.0-fold, 2.0-fold, 1.9-fold and 2.1-fold, respectively, (*p*<0.05 for all), whereas FXR protein expression was markedly decreased to 56% of controls (*p*<0.05, Fig. 5B and C). Moreover, we observed that the protein levels of nuclear receptors PPARα, HNF1α, LXR and SHP and transcription factors HNF3β, NRF2 and AHR were not significantly altered (Fig. 5B and C). The up-regulation of nuclear receptors VDR, HNF4α and RARα and the down-regulation of nuclear receptor FXR in the nuclei of human obstructive cholestatic hepatocytes were further confirmed by immunofluorescence microscopy labeling with specific antibodies (Fig. 5D). Moreover, as shown in supplementary S1 Fig., elevated PXR mRNA levels (1.7-fold) were observed as described previously [7].

### Positive and significant correlations between the reduced levels of nuclear receptor FXR and reduced detoxification enzymes and canalicular membrane transporters in human obstructive cholestatic livers

**Fig 6:**
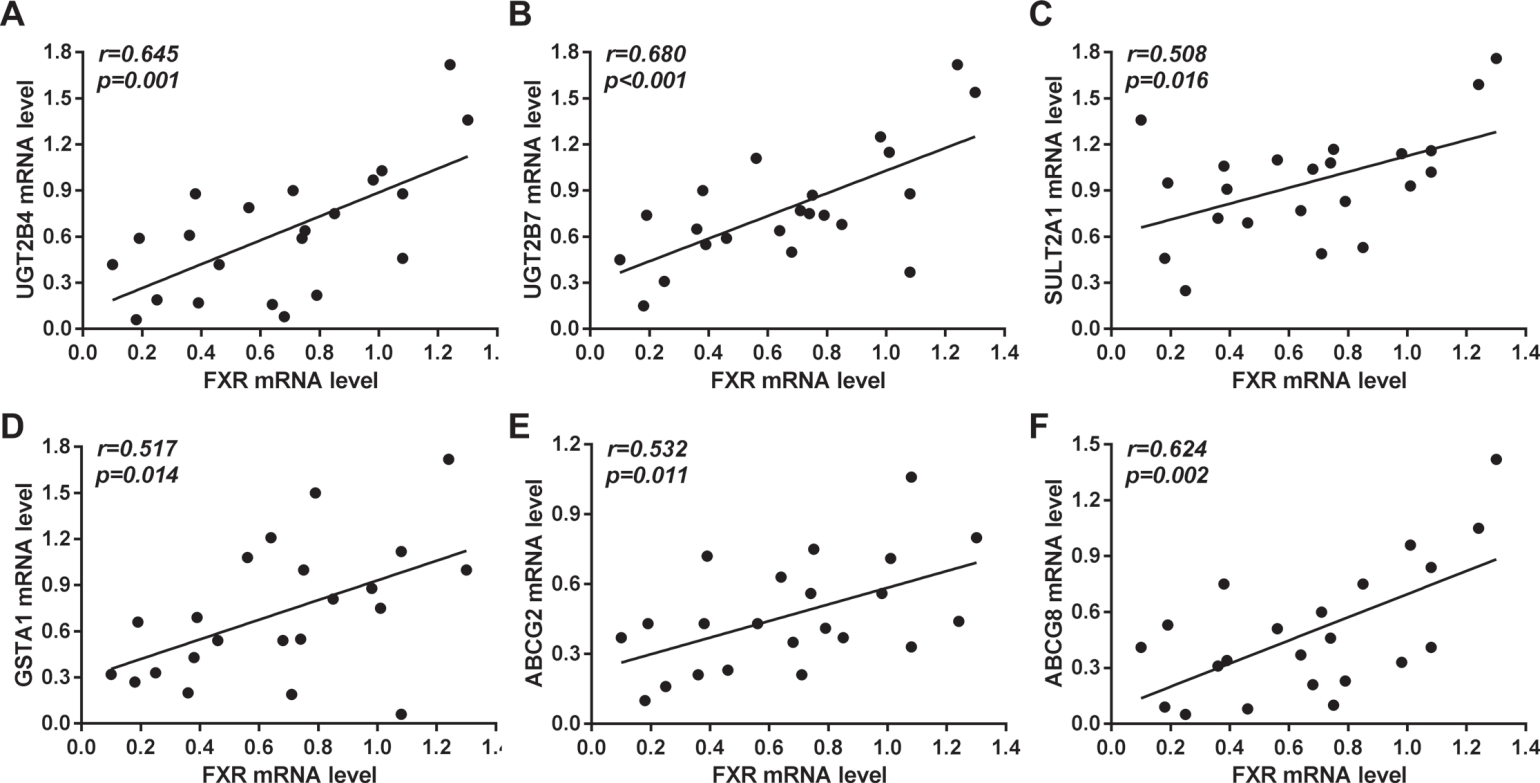
Correlation of the mRNA levels of the nuclear receptor, FXR, with the mRNA levels of detoxification enzymes and canalicular membrane transporters in human obstructive cholestatic livers. The mRNA levels of bile acid receptor FXR and detoxification enzymes (A) UGT2B4, (B) UGT2B7, (C) SULT2A1, (D) GSTA1, and canalicular membrane transporters (E) ABCG2, and (F) ABCG8 in the livers of patients with obstructive cholestasis due to gallstone biliary obstruction (n = 22). The data were analyzed by the Pearson’s correlation test using GraphPad Prism 6.01. *r*, Pearson correlation. A value of *p* < 0.05 was considered to be statistically significant.

As expected, there were positive and significant correlations between the reduction in levels of hepatic FXR mRNA and the decrease in the levels of detoxification enzymes UGT2B4, UGT2B7, SULT2A1 or GSTA1 in obstructive cholestatic patients (*r* = 0.645, *p* = 0.001; *r* = 0.680, *p*<0.001; *r* = 0.508, *p* = 0.016; and *r* = 0.517, *p* = 0.014, respectively) (Fig. 6A-6D). However, there was no correlation with the reduction in GSTA2–5 and GSTM1–5 mRNA levels (data not shown). As indicated in Fig. 6E and F, FXR down-regulation was positively correlated with canalicular membrane ABCG2 and ABCG8 mRNA levels (*r* = 0.532, *p* = 0.011, and *r* = 0.624, *p* = 0.002, respectively), but not with ABCG5 mRNA levels (data not shown). These results demonstrated that down-regulation of FXR may decrease the expression of detoxification enzymes and canalicular membrane transporters in human obstructive cholestasis.

## Discussion

The synthesis, transport and metabolism of bile acids are significantly altered in cholestasis [1–3]. We have previously reported that the expression of MDR3, MRP3, and MRP4 were increased whereas NTCP and CYP7A1 were decreased in human obstructive cholestasis originating from gallstone blockage of bile ducts [6, 7]. In the current study, we demonstrated that the hepatic expression of bile acid synthetic enzymes CYP7B1 and CYP8B1, and basolateral membrane transporters OSTβ and OCT1 were up-regulated. However, the expression of detoxification enzymes UGT2B4/7, SULT2A1, GSTA1–4 and GSTM1–4 and canalicular membrane transporters ABCG2 and ABCG8 were significantly down-regulated in obstructive cholestatic patients, and were significantly and positively correlated with FXR reduction. These data suggested that targeted down-regulation of FXR may improve the hepatic detoxification ability in human obstructive cholestasis.

The bile acid receptor FXR exerts a crucial role in mediating the hepatic expression of bile acid detoxification enzymes, synthetic enzymes, and membrane transporters [3, 14, 15]. The bile acid synthetic enzyme, Cyp7a1, has been shown to be repressed by the induced expression and activity of nuclear receptors FXR/SHP and plasma Fgf15 in cholestatic rodents [24–26]. In contrast to FXR/SHP induction in rodent cholestasis [1–3], FXR expression was found to be reduced without changes in SHP expression in liver samples of patients with obstructive cholestasis. These results indicate that elevated plasma FGF19 originating from the intestine may contribute to the repression of CYP7A1 expression in human obstructive cholestasis, but not to the hepatic nuclear receptors FXR/SHP. This is supported by the finding that plasma and hepatic FGF19 levels were elevated in extrahepatic cholestasis [12, 26–28]. Moreover, the induced modulators HNF4α and LRH-1 may also be involved in CYP7A1 down-regulation in human obstructive cholestasis. Significantly positive correlations have been found between nuclear receptor FXR levels, and decreased levels of the detoxification enzymes UGT2B4/7 (glucuronidation), SULT2A1 (sulphation), and GSTA1 (glutathione conjugation) in patients with obstructive cholestasis. Increased CYP2B6 expression, as an activation index of nuclear receptor CAR, implies that increased nuclear CAR protein with unchanged mRNA levels may be due to effects on nuclear translocation in human obstructive cholestasis as described previously [7]. Furthermore, elevated PXR and VDR levels were also observed in the present study, and a previous study [7]. Thus, we speculate that FXR reduction may be at least partially responsible for the down-regulation of detoxification enzymes UGT2B4/7, SULT2A1 and GSTA1 in human obstructive cholestatic liver because the nuclear receptors, VDR, PXR and CAR, are also their modulators [3, 14, 29]. However, induction of liver VDR and reduction of SULT2A1 in human obstructive cholestasis, were paradoxically observed. Because VDR induces not only detoxification enzymes (e.g. SULT2A1), but also bile acid transporters (e.g. MRP3) [3, 14, 29], the paradoxical results may be due to the different roles played by VDR in various diseases and species. In human cholestasis, elevated VDR may up-regulate levels of bile acids transporters (e.g. MRP3), but not induce detoxification enzymes (e.g. SULT2A1). Another possible explanation is that in order to increase detoxification enzyme levels, including PXR and CAR, VDR may need the cooperation of other modulators that may be decreased in human obstructive cholestasis. Previous studies have demonstrated that the detoxification enzymes UGTs, SULTs, and GSTs, involved in the detoxification of xenobiotics and endobiotics (e.g. bile acids and drugs), protect hepatocytes against toxicant-induced damage [1, 3, 14]. The present results suggest that hepatic detoxification ability may be greatly impaired due to a dramatic reduction of most detoxification enzymes expression in human obstructive cholestatic patients.

Furthermore, the upregulation of bile acid synthetic enzymes CYP7B1 and CYP8B1 may be attributed, at least in part, to FXR reduction, which is associated with increased expression of their suppressors HNF4α and LRH-1 in human obstructive cholestatic liver [2, 6, 14]. Their up-regulation during cholestasis indicates that the alternative pathway of bile acid synthesis was activated, directing the bile acid synthesis towards to cholic acid (CA), but not chenodeoxycholic acid (CDCA). This up-regulation may play a protective role, considering that that CYP8B1 deficiency in mice led to the production of CDCA over CA [30, 31]. CDCA is known to be more hepatotoxic than CA [3, 30, 31]. This may also explain the clinical phenomenon of plasma total CA/CDCA > 1.0 in patients with obstructive cholestasis [31]. Moreover, the induced CYP8B1 expression may correlate with the development of gallstones in human obstructive cholestasis due to gallstone blockage of bile ducts because gallstone formation has been shown to be averted with depletion of CA in mice [30].

Moreover, FXR repression may cause a reduction in canalicular membrane transporters ABCG2 and ABCG8 in human obstructive cholestatic liver because there were significantly positive correlations between the expression of FXR and ABCG2 or ABCG8, and FXR is known to modulate their levels in rodent liver and hepatocytes [3, 14]. Recent studies have reported that Abcg5 deficient mice were more susceptible to cholestasis compared to normal, and that ABCG2 serves as a bile acid and cholesterol transporter [32, 33]. Thus, the down-regulation of ABCG2 and ABCG8 may involve in the disruption of bile acid and cholesterol homeostasis during cholestasis. Although FXR stimulates the expression of OSTα/β in cholestatic rodents and hepatoma cell lines [3, 14], the down-regulation of hepatic OSTα/β mRNA in human obstructive cholestasis was not observed in parallel with the FXR reduction. In contrast, mRNA and protein levels of OSTβ were significantly increased, whereas OSTα protein levels were decreased without changes in transcription. The repression of OSTα protein levels may play a protective role in human obstructive cholestasis as suggested by the fact that Ostα deficient mice exhibited decreased liver injury and cholestasis in response to common bile duct ligation [34, 35]. In addition, we observed up-regulation of OCT1 protein levels at the basolateral membrane of hepatocytes in patients with obstructive cholestasis, which differs from the observations in rodent cholestasis, and some types of human cholestasis [36–38]. However, the specific molecular mechanism and the role of OCT1 regulation in human obstructive cholestasis needs to be further studied. Although we have demonstrated that down-regulation of detoxification enzymes and canalicular membrane transporters may be mediated by the FXR reduction in human obstructive cholestasis, direct evidence supporting this conclusion should be addressed in future studies.

In summary, we have demonstrated that hepatic detoxification enzyme levels were significantly reduced in human obstructive cholestasis due to gallstone biliary obstruction. This was positively correlated with a reduction nuclear receptor, FXR, levels. Targeting bile acid receptor FXR by agonists (e.g. INT747) may be a potential therapy to improve liver detoxification ability and attenuate liver injury in human obstructive cholestasis.

## Supporting Information

S1 Fig Liver PXR and CAR mRNA levels in human obstructive cholestasis. Real-time qPCR analysis of nuclear receptors mRNA levels (A) PXR and (B) CAR from control livers (n = 22) and obstructive cholestatic livers (n = 22). *p < 0.*01* versus controls. A value of *p* < 0.05 was considered to be statistically significant.
(TIF)

S1 Table Sense and antisense primers used for Real-Time qPCR (SYBR Green). Real-time quantitative polymerase chain reaction (qPCR), using a SYBR premix Ex Taq II kit (Takara Biotechnology, Tokyo, Japan), was performed in a Bio-Rad CFX96 real-time system machine (Bio-Rad, Hercules, CA) to determine the mRNA levels of specific genes, whose primers are listed in the Table. Real-time qPCR (SYBR Green) primers were designed by primer premier 5.0 (PREMIER Biosoft, Palo Alto, CA). The primers were used for analysis only if their amplification efficiency was more than 90%, and the melt curve was acceptable.
(DOC)
