# Supplementary figures and images for: Hepatic Expression of Detoxification Enzymes Is Decreased in Human Obstructive Cholestasis Due to Gallstone Biliary Obstruction
Source: PLoS One. 2015 Mar 23;10(3):e0120055. doi: 10.1371/journal.pone.0120055 (PMC4370735; doi:10.1371/journal.pone.0120055)

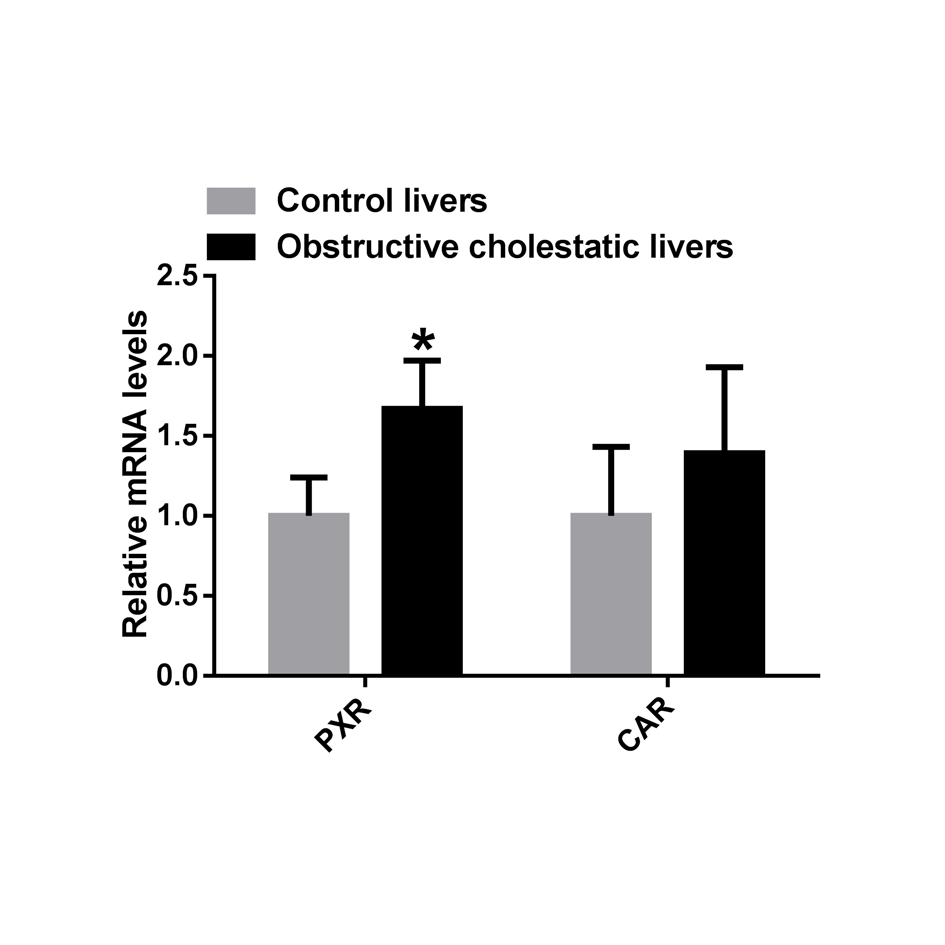

Supplement: S1 Fig — Real-time qPCR analysis of nuclear receptors mRNA levels (A) PXR and (B) CAR from control livers (n = 22) and obstructive cholestatic livers (n = 22). *p < 0.01 versus controls. A value of p < 0.05 was considered to be statistically significant. (TIF) [file pone.0120055.s001.tif]
