# Supplementary material for: Hepatic Expression of Detoxification Enzymes Is Decreased in Human Obstructive Cholestasis Due to Gallstone Biliary Obstruction
Source: PLoS One. 2015 Mar 23;10(3):e0120055. doi: 10.1371/journal.pone.0120055 (PMC4370735; doi:10.1371/journal.pone.0120055)
Supplement: S1 Table — Real-time quantitative polymerase chain reaction (qPCR), using a SYBR premix Ex Taq II kit (Takara Biotechnology, Tokyo, Japan), was performed in a Bio-Rad CFX96 real-time system machine (Bio-Rad, Hercules, CA) to determine the mRNA levels of specific genes, whose primers are listed in the Table. Real-time qPCR (SYBR Green) primers were designed by primer premier 5.0 (PREMIER Biosoft, Palo Alto, CA). The primers were used for analysis only if their amplification efficiency was more than 90%, and the melt curve was acceptable. (DOC) [file pone.0120055.s002.doc]

**S1 Table. Sense and Antisense Primers Used for Real-Time qPCR (SYBR Green)**

| **Gene** | **Accession No.** | **Sense Primer (5ˊ→3ˊ)** | **Antisense Primer (5ˊ→3ˊ)** | **Products(bp)** |
| --- | --- | --- | --- | --- |
| CYP7B1 | NM_004820.3 | ggagcacatcatttaggctttc | ccctttcttttgacctgttgac | 156 |
| CYP8B1 | NM_004391.2 | ccctctttccctacctctcagt | aagtgtgtgaccataagcagga | 235 |
| CYP2B6 | NM_000767.4 | gagaccaccagcaccactct | ggccaatcacctgttcaatc | 100 |
| CYP27A1 | NM_000784.3 | ctcactctatgccaccttcctc | cattccaaccatccaggtatc | 80 |
| CYP3A4 | BC101631.1 | agtggaaaactcaaggagatgg | cgatgttcactccaaatgatgt | 166 |
| UGT2B4 | NM_021139.2 | cctatgtgcctgttgttatgtca | aacatttggtaagagtgggtgag | 248 |
| UGT2B7 | NM_001074.2 | ctctggggtcaatggtcagta | ccacttgtagagccgagtattg | 146 |
| SULT2A1 | NM_003167.3 | aacaggacacaggaagaaccat | cagtccccagatacaccttttc | 199 |
| GSTA1 | NM_145740.3 | aatggttgagattgatgggatg | gggctctctcctttatgtctttc | 104 |
| GSTA2 | NM_000846.4 | ccttcttctgccctttagtcaa | gtagtcttgtccgtggctcttt | 124 |
| GSTA3 | NM_000847.4 | tgaaaaccagaatcagcaacct | acaacaggcacaatcaacactt | 215 |
| GSTA4 | NM_001512.3 | gagtccgtgagatgggttttag | ttggaacagcaggtggttac | 112 |
| GSTA5 | NM_153699.1 | tatgtcaaccagaggaaagagatg | ttcaaaggcagggaagtagc | 80 |
| GSTM1 | NM_000561.3 | ctacttgattgatggggctcac | atggtctggttctccaaaatgt | 132 |
| GSTM2 | NM_000848.3 | accttccttcctgttagtg | tcaatgctgctccttcat | 92 |
| GSTM3 | NM_000849.4 | gctcacctttgtggattttctc | tgtaagtaggcagcgattttctc | 142 |
| GSTM4 | NM_000850.4 | actttcccaatctgccctactt | tagcctggttctccaaaatgtc | 147 |
| GSTM5 | NM_000851.3 | cttagtggatgggtgtgtgtgt | acagtgaggaacagagggatgt | 109 |
| OCT1 | NM_003057.2 | tcctcttcctgctctacta | ttcggtgacatcctcttc | 167 |
| ABCG2 | NM_004827.2 | gtttatccgtggtgtgtctgg | tgattgttcgtccctgcttag | 173 |
| ABCG5 | NM_022436.2 | tctgcctacaagaaatcagcaa | gaacgaagaaaaggaggaacaa | 236 |
| ABCG8 | NM_022437.2 | caatgccctctacaactccttc | atcttcatcagcccttcaaaac | 129 |
| VDR | NM_000376.2 | cagaaggagaaggaagga | tgaggcaacagcattatc | 121 |
| PPARα | NM_001001928.2 | gaaatgggaaacatccaagaga | cacaggataagtcaccgagga | 168 |
| HNF1α | NM_000545.5 | gatgagctaccaaccaagaagg | cctattgcactcctccactagc | 141 |
| HNF4α | NM_000457.3 | acctcaaagccatcatcttctt | gtcatactggcggtcgttg | 125 |
| RARα | NM_000964.3 | ctgggcaaatacactacgaaca | taatgatgcacttggtggagag | 100 |
| LXR | NM_005693.3 | ttcgcaaatgccgtcagg | gccgcttcagtttcttcagg | 81 |
| FXR | NM_001206979.1 | cctcctcacctcattgtctc | acctgccacttgttctgtta | 442 |
| SHP | NM_021969.2 | agaatatgcctgcctgaa | tggtcggaatggacttga | 196 |
| HNF3β | NM_021784.4 | tctccatcaacaacctcatgtc | agtacaccccctggtagtagga | 222 |
| NRF2 | NM_006164.4 | agcgacggaaagagtatgagc | acctgggagtagttggcagat | 193 |
| AhR | NM_001621.4 | aagtctcccttcataccttcaga | gtgatgttgctgttgctgttct | 108 |
|  | | | | |
